# Unicompartmental knee arthroplasty in the over 70 population

**DOI:** 10.1186/1471-2482-13-S1-A42

**Published:** 2013-09-16

**Authors:** D Rosa, G Balato, G Ciaramella, SL Di Donato, A Di Meo, C Andolfi

**Affiliations:** 1 Department of Orthopaedic Surgery, Federico II University, Naples, Italy

## Introduction

Unicompartmental prosthesis (UKA) is a viable option for the treatment of unicompartmental osteoarthritis, most often confined to the medial compartment. Appear to have been well codified indications, contraindications, and surgical techniques related to unicompartmental arthroplasty. The data in the literature show that the selection criteria can be divided into general origin factors (age, weight, activity level) and local factors, more closely related to the joint to be treated [1,2]. The patient's age over 65 years and the functional requirements of a sedentary activity comparable to justify its use in elderly patients with clinical signs and imaging involvement of only one of the joint compartments [3]. We agree, however, with those who recommend its use in older subjects, since it is well tolerated, with low morbidity, reduced blood loss, better preservation of the joint and saving of bone tissue. Other advantages include lower costs, shorter hospitalization, faster rehabilitation and conferment of a greater than pre-op functional efficiency that fully meets the needs of elderly patients. The authors report their experience in 12 patients treated surgically implanting a unicompartmental prosthesis.

## Materials and methods

We evaluated 12 unicompartmental tibiofemoral joint prosthesis (2 lateral and 10 medial) from 2010 to 2012. Of all patients (7 F and 5 M), mean age 72 years (range 57-88), we analyzed in the pre-operative, the of lower limb alignment on an examination of full standing radiographs.

We considered it appropriate to perform radiographs in 45 ° of flexion under load (projection Rosenberg) and axial patella (Merchant view) to exclude the involvement of other joint compartments. For the evaluation of the clinical results we used the Knee Society Score (KSS), the Womac score and the SF-36 for quality of life. All the patients were operated with a medial approach for the medial UKA implants and a lateral approach for the lateral UKAs. A tourniquet was used in all cases except one patient with severe arterial vessel disease. All patients received antibiotic treatment (second generation cephalosporin) and an anticoagulant treatment (low-molecular-weight heparin).

## Results

In all patients, we prescribed a standard rehabilitation protocol. Range of motion was satisfactorily increased. The mean flexion was 110 degrees with significant improvement in pain symptoms joint. Pain at extreme ROM values was also reduced. Improvement was most evident during walking: increase in the free perimeter and a decrease in pain intensity. The subjective result was very satisfactory for 7 patients (58%), satisfactory for 4 patients (34%), unsatisfactory in 1 case (8%).

## Discussion and conclusion

The unicompartmental prosthesis for the treatment of both medial and lateral overload has reached a considerable degree of reliability mainly due to the experience of the surgeon and to indications. The unicompartmental prosthesis therefore stands as an alternative surgical solution to total knee arthroplasty offering some definite advantages among which are the preservation of bone stock, minimally invasive surgery, lower costs and shorter hospitalization[1,2,4]. From the point of view of functional recovery rehabilitation is faster with good results already in the immediate post-operative.
